# Unveiling the role of erinacines in the neuroprotective effects of *Hericium erinaceus*: a systematic review in preclinical models

**DOI:** 10.3389/fphar.2025.1582081

**Published:** 2025-06-23

**Authors:** E. T. Spangenberg, A. Moneypenny, G. G. Bozzo, M. L. Perreault

**Affiliations:** ^1^ Department of Biomedical Sciences, University of Guelph, Guelph, ON, Canada; ^2^ Department of Molecular and Cellular Biology, University of Guelph, Guelph, ON, Canada; ^3^ Department of Plant Agriculture, University of Guelph, Guelph, ON, Canada

**Keywords:** erinacine, *Hericium erinaceus*, neuroprotection, neuroinflammation, cognitive function, neurodegenerative diseases

## Abstract

The medicinal mushroom lion’s mane (*Hericium erinaceus*) is suggested to have therapeutic potential for neurological disorders due to its neuroprotective and neurotrophic properties. Mycelia of *H*. *erinaceus* contain erinacines, a group of cyathane diterpenoids, however no systematic review has explored the broader role of these compounds in mediating the neurobiological effects of the mushroom. This systematic review was therefore performed to enhance the depth of understanding surrounding the neurobiological impact of the various erinacine compounds using various cellular and rodent models. A secondary focus was to assess how study outcomes were influenced by the chemical complexity of the administered treatments. The Preferred Reporting Items for Systematic Reviews and Meta-Analyses (PRISMA) guidelines were utilized. The findings showed the broader potential of *H. erinaceus* mycelial formulations, and their derived erinacines, to exert dose-dependent benefits in motor, cognitive, and depression-like behaviours in animal models. Synthesis of records highlighted the ability of both erinacines and *H. erinaceus* to induce antioxidant responses and activate pro-survival signaling pathways. However, erinacine A and C uniquely induced the accumulation of the transcription factor Nrf2, a key regulator of the antioxidant response. These erinacines were also anti-inflammatory, enhanced neurogenesis and cell survival, and improved cognitive and behavioral outcomes *in vivo*. These findings suggest the promise of *H. erinaceus* extracts and individual erinacines as accessible, cost-effective interventions for aging-related and neurodegenerative conditions.

## Graphical Abstract

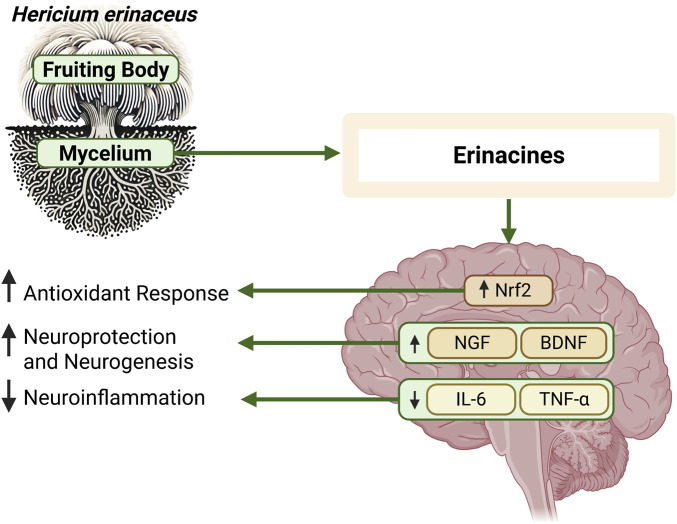

## Introduction

Natural therapies have become increasingly popular in recent years as there is a growing list of medicinal plants and mushrooms containing both preventative and restorative neuroprotective compounds (Venturella et al., 2021). Early clinical trials have demonstrated the effectiveness of many medicinal mushrooms for the treatment and management of various diseases including Alzheimer’s Disease (AD), Parkinson’s Disease, depression, anxiety and sleep disorders (Rai et al., 2021; Venturella et al., 2021; Pepe et al., 2023). The therapeutic effects of medicinal mushrooms are often associated with some primary compounds, such as fatty acids and sterols, and secondary metabolites including various terpene/terpenoid compounds (Łysakowska et al., 2023).

Some medicinal mushrooms, such as the agaricomycete Lion’s mane (*Hericium erinaceus* (Bull.) Pers.), are vital components of traditional Asian medicine (Spelman et al., 2017) and are often used as ingredients in nutraceutical products (Niego et al., 2021). Lion’s mane mushroom, also termed bearded tooth fungus, has garnered interest for its applications in brain health as a nootropic, or “smart drug” (Onaolapo et al., 2019). Nootropics are a heterogenous group of medicinal substances often derived from plants and other organisms that are believed to improve human learning, memory and cognition (Malík and Tlustoš, 2022). The nootropic effects of *H. erinaceus* were demonstrated in early clinical trials, improving measures in assessments of cognitive ability in both young (19–45) and older (>55) healthy adults (Docherty et al., 2023; Černelič Bizjak et al., 2024) as well as in older adults with mild cognitive impairments (Mori et al., 2009). The efficacy of nootropics is notably increased in cases of learning and memory impairments, where they can serve as potential therapeutics (Schifano et al., 2022).

*Hericium erinaceus* mushrooms grow primarily on wood-based substrates (Onaolapo et al., 2019). *Hericium erinaceus* consists of an external fleshy fruiting body that develops from a substrate bound mycelial structure. Both the mycelium and fruiting body of *H. erinaceus* have neuroprotective properties, although these tissues differ in their composition of bioactive molecules (Corana et al., 2019). *H. erinaceus* mycelia are rich sources of erinacines, a class of cyathane diterpenoids with fused 5-carbon, 6-carbon, and 7-carbon ring structures, whereas hericenones are a class of meroterpenoids found exclusively within fruiting bodies of this basidiomycete (Corana et al., 2019). Other secondary metabolites that occur in *H. erinaceus* include hericerins, isoindoline-1-ones, and erinaceolactones (Friedman, 2015). Many of these bioactive constituents exhibit anti-carcinogenic, anti-aging, anti-inflammatory, and/or antioxidative properties. Some erinacines and hericenones easily cross the blood brain barrier via passive diffusion, where they function as neurotrophin-stimulating compounds (He et al., 2017; Li et al., 2018). These neurotrophins, or growth factors, such as nerve growth factor (NGF), brain-derived neurotrophic factor (BDNF) and neurotrophin-3 (NT-3), activate the tyrosine receptor kinase (Trk) family of receptors to promote neuronal survival, plasticity, and repair (Saragovi et al., 2019). To date it is known that hericenones A-H as well as erinacines A-C, H, and I stimulate synthesis of NGF *in vitro*, but can only do so when administered to glial cells and cannot induce NGF synthesis in neurons alone (Kawagishi et al., 1994; Phan et al., 2014). In addition, erinacine A attenuates neurotoxicity through the upregulation of pro-survival pathways, while decreasing activity of pro-apoptotic pathways (Lee et al., 2020).

Although there have been integrative reports describing *H. erinaceus* in therapeutic applications, relatively few studies have addressed the unique contribution of individual erinacines to these effects. As such, this review aims to narrow this gap by examining the cumulative literature on the neurobiological effects of *H. erinaceus* mycelia and, at the same time, provide insights into the molecular actions of specific erinacines and their contribution to the therapeutic profile of *H. erinaceus*.

## Methods

The current systematic review abided by the Preferred Reporting Items for Systematic Reviews and Meta-Analysis (PRISMA) guidelines. Two distinct literature searches were conducted independently by two individuals screening the Web of Science, PubMed and ScienceDirect databases in February 2024 using three strings of search criteria all joined by Boolean commands. The key words consisted of either “erinacine” or “*Hericium erinaceus*” and (“Neuroprotection” or “Neurotrophins” or “Neuron” or “Neuronal System”). Primary data, free text available, preclinical experimental studies (*in vivo* and *in vitro*) published in English in a peer-reviewed journal up to July of 2024 and that also evaluated individual erinacine compounds or *H. erinaceus* mycelia that reported erinacine concentrations with a neurological focused cellular, molecular, and/or behavioural outcome were included. Exclusion criteria were adopted based on studies that mainly focused on fruiting body administrations, bioavailability, compound isolation, molecular chemistry, cancer research models, non-neurologic outcomes, no reporting of erinacine content, and analysis of alternate *Hericium* species. Retracted articles, reviews, meta-analyses, conference abstracts, book chapters, and unpublished results were additionally excluded from the analysis. The search initially returned 66 and 123 reports in the erinacine and *H. erinaceus* searches, respectively, which was reduced to 46 and 107 reports after removal of duplicates. Further screening was then conducted to assess records suitability for inclusion and, after removal of unrelated studies and additional records identified from references, 26 and 66 full-text articles underwent independent screening. Following screening, additional studies were removed after not meeting the inclusion criteria, many of which did not report erinacine concentrations.

## Results

**FIGURE 1:**
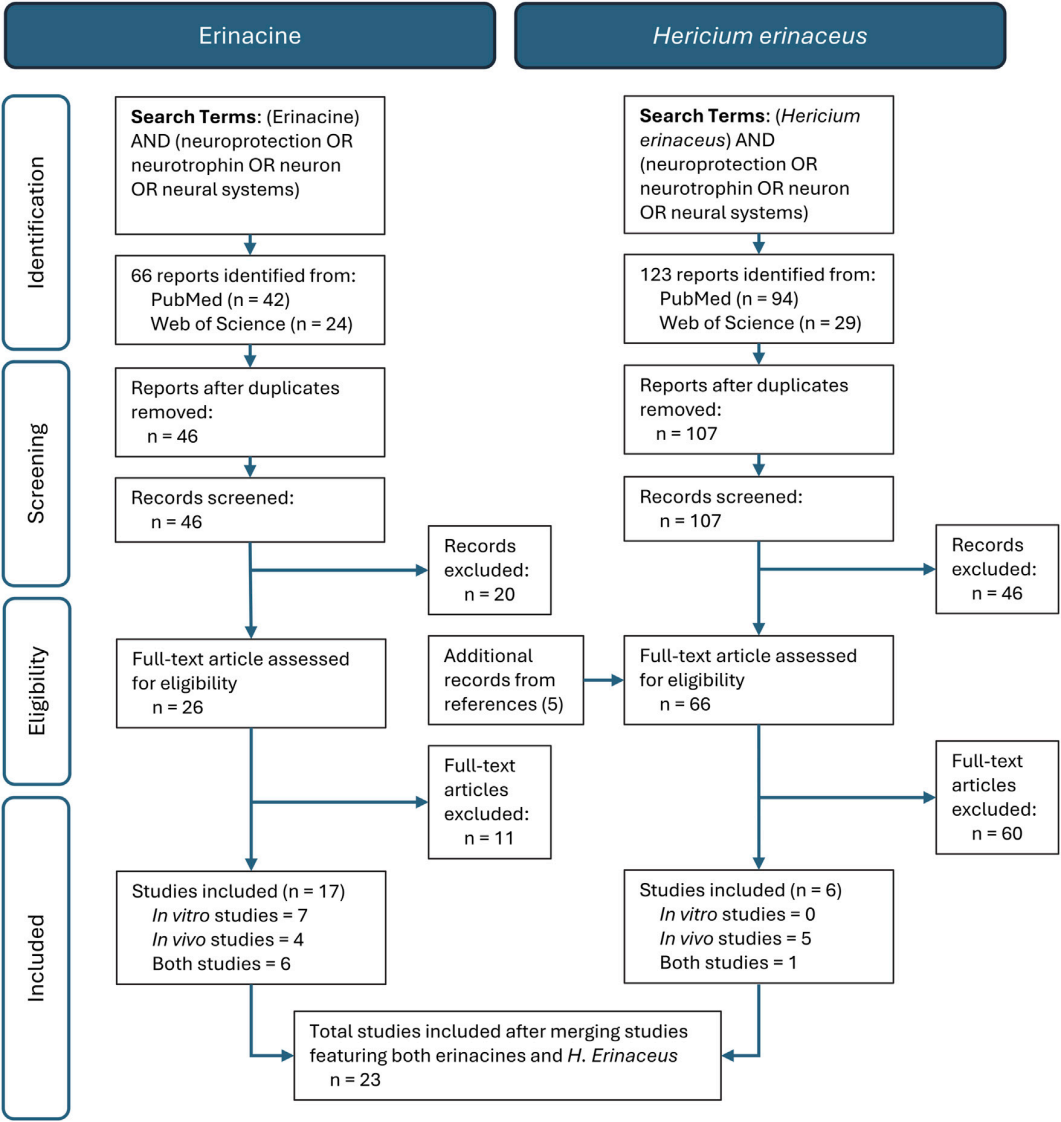
PRISMA Workflow Diagram of a systematic review of the *in vitro* and *in vivo* studies related to the neurobiological effects of *Hericium erinaceus* mycelium whole extracts and the erinacines.

After the final synthesis of studies across all searches there were a total of 23 studies included in the review (Figure 1) that were allocated to *in vitro* or *in vivo* categories, each of which included either individual erinacines or some type of *H. erinaceus* mycelial preparation. A total of seven studies investigated treatment outcomes of both *in vivo* and *in vitro* analyses. Nine of the included studies evaluated solely *in vivo* outcomes, whereas seven studies evaluated solely *in vitro* outcomes.

Various types of *H. erinaceus* mycelial formulations and erinacine compounds were assessed in both *in vitro* (Table 1) and *in vivo* (Table 2) studies. For the erinacine studies, erinacine A was the most investigated compound, with its biological effects assessed in twelve out of the seventeen *in vitro* and *in vivo* studies. Erinacine C and erinacine S were the test compounds in seven and six reports, respectively. Erinacine C was mostly evaluated *in vitro*, with only two of the seven papers featuring *in vivo* experiments. The erinacines B, E, F, L, Z1 and Z2 appeared in one record each and therefore only erinacine A, erinacine S and erinacine C were critically evaluated in this review. Six studies that administered *H. erinaceus* mycelia (HEM) or mycelial extracts (HEME) reported the concentration of erinacine A found within the fungus. All were *in vivo* studies, with only one of these reporting *in vitro* analyses. Three studies administered HEM, two studies administered ethanolic HEME, and one study administered both HEM and HEME.

**TABLE 1:** Characteristics of the included *in vitro* studies following PRISMA guidelines.

Author	Treatment	Focus	Experimental model	Findings
Hsu et al. (2022) ^a^	EA	Neuroprotection, astrocyte GLT-1 function, glutamate homeostasis	Oxygen glucose deprived mouse CTX glia-neuron co-culture	Preserved astrocyte enriched GLT-1 function to maintain glutamate homeostasis
Huang et al. (2021)	EA, ES, EC	Oligodendrocyte differentiation	Rat CTX OPCs; cerebellar slice cultures	EA & ES: ↑ myelin basic protein expression, ↑ # of mature oligodendrocytes
Lee et al. (2024) ^a^	EC	Gene expression, calcium signaling, oxidative stress, Nrf2	LPS-induced mouse whole brain mixed-glia cultures and BV-2 cells	Nrf2 pathway mediated protection against neuronal injury and microglial activation
Lee et al. (2020) ^a^	EA	Parkinson’s disease pathology	MPP + -treated mouse N2a cells or substantia nigra neuron culture	Prevented dopaminergic degeneration and motor dysfunctions through cell survival pathway promotion
Lee et al. (2022) ^a^	EA	Neuroinflammation	LPS and/or IFN-γ-induced BV-2 cells, CTX TNA2 cells, N2a cells treated with LPS-treated BV-2 conditioned medium	Inhibited expression of proinflammatory factors involved in activation of glial cells. Suppressed cell death pathways
Lin et al. (2024)	EA, EC, ES	Neuroprotection and immunomodulation	LPS-induced BV-2 microglia cells and SH-SY5Y co-cultures	Induces neuroprotection and reduction of inflammation
Lin et al. (2023)	ES	Neurite outgrowth and regeneration, gene expression	Mouse CTX neuron culture, rat dorsal root ganglion culture	Enhanced neurite outgrowth and post-injury axon regeneration
Rascher et al. (2020)	EC	Transcriptional activation and neurotrophin signaling	PC12 cells cultured with 1321N1 (astrocyte cells) conditioned medium	Enhances expression of NGF and BDNF in glial cells
Rupcic et al. (2018)	EA, EB, EC, EZ1, EZ2	Metabolic profiles, neurotrophin expression	PC12 cells with or without 1321N1 cell conditioned medium	Stimulated neurotrophin production in astrocytic cells
Wang et al. (2019)	EC	Neuroinflammation and cell signaling	LPS-induced BV2 microglial cells	Involve activation of the Nrf2/HO-1 pathway and inhibition of IκB and iNOS expressions
Wei et al. (2023)	EA, EL, EC, EF	Neurotrophic activity and anti-neuroinflammation	PC12 cells, LPS-induced BV2 microglial cells	Showed neurotrophic activity in PC-12 cells and limited NO production in BV2 cells
Wu et al. (2023) ^a^	HEME – [EA]: 5 mg/g	Neuroprotection	tBH-induced human neuroblastoma SK-N-SHMJD78 cells and SCA3 cells	↑ lifespan, ↓ apoptosis via downregulation of p53 and NF-kB
Yang et al. (2020) ^a^	EA, ES	Purinoceptor signaling	Human neuroblastoma SH-SY5Y cells, human osteosarcoma cells	ES inhibits ATP-induced rise in [Ca2+] in P2R-mediated signal transduction
Zhang et al. (2017)	EA	Neuroprotection and neurite growth	PC12 cells, rat CTX neuron cultures	↑ NGF-mediated neurite outgrowth through TrkA and Erk1/2

^a^ Indicates studies included in both *in vitro* and *in vivo* results.

Abbreviations: CTX, cortex; EA, erinacine A; EB, erinacine B; EC, erinacine C; EF, erinacine F; EL; erinacine L; ES, erinacine S; EZ1, erinacine Z1; EZ2, erinacine Z2; GLT-1, glutamate transporter 1; HE, *Hericium erinaceus*; HEME, *Hericium erinaceus* mycelia erinacine extracts; HO-1, heme oxygenase-1; H_2_O_2_, hydrogen peroxide; Hip, hippocampus; IFN-γ, interferon-γ; iNOS, inducible nitric oxide synthase; IκB, inhibitor of NF-κB; LPS, lipopolysaccharide; MPP+, 1-methyl-4-phenylpyridinium; NF-κB, nuclear factor kappa-light-chain-enhancer of activated B cells; NO, nitric oxide; Nrf2, nuclear factor erythroid 2–related factor 2; OPCs, oligodendrocyte precursor cells; P2R, purinoceptor; SCA3, Spinocerebellar ataxia type 3; tBH, *tert*-butyl hydroperoxide; TrkA, tropomyosin receptor kinase A.

**TABLE 2:** Characteristics of the included *in vivo* studies following PRISMA guidelines.

Author	Treatment	Focus	Animal species/Model	Findings
Chen et al. (2016)	EA, ES	Alzheimer’s disease	APP/PS1 mice	↓ Aβ plaque burden, ↑ cortical insulin-degrading enzyme levels
Chiu et al. (2018)	HEME – [EA]: 5 mg/g	Depression	Restraint stress, ICR mice	Normalization of depressive behaviour, ↑ BDNF and modulation of PI3K/Akt/GSK-3β pathway
Hsu C.-H. et al. (2023)	HEM – [EA]: 30 μg/g	Parkinson’s disease	MPTP-treated C57BL/6 mice	Amelioration of oxidative stress, ↑ dopamine and tyrosine hydroxylase levels
Hsu C.-L. et al. (2023)	EA	Traumatic optic neuropathy	Traumatic optic neuropathy, Wistar rats	Neuroprotection and preservation of visual function
Hsu et al. (2022) ^a^	EA	Ischemia	tHI-induced C57BL/6 mice	Preserved astrocyte-enriched GLT-1 function to maintain glutamate homeostasis
Huang et al. (2021)	EA, EC, ES	Oligodendrocyte maturation	*Ex vivo* cerebellar slice of SD rats	EA & ES: ↑ myelin basic protein expression, ↑ # of mature oligodendrocytes
Lee et al. (2024) ^a^	EC	TBI	TBI-induced SD rats	Nrf2 pathway mediated protection against neuronal injury and microglial activation
Lee et al. (2020) ^a^	EA	Parkinson’s disease	MPTP-treated C57BL/6 mice	Prevented dopaminergic degeneration and motor dysfunctions through cell survival pathway promotion
Lee et al. (2014)	EA	Ischemia and reperfusion injury	Global ischemia, SD rats	Neuroprotection and free radical scavenging related to endoplasmic reticulum stress signaling
Lee et al. (2022) ^a^	EA	LPS-induced inflammation in microglia	SD rats	Prevention of motor dysfunction and ↓ microglia-mediated neuroinflammation
Lee et al. (2021)	HEM – [EA]: 5 mg/g	Neuroprotective effects on aging	SAMP8 mouse model of aging	↑ learning and memory through ↓ oxidative stress and ↓ amyloid aggregation
Li et al. (202)	HEM – [EA]: 7.2 mg/g	Sleep disruption	Sleep disturbed C57BL/6 mice	Reversed sleep disturbances and ↓ anxiety-like behaviour
Tsai-Teng et al. (2016)	HEM, HEME – [EA]: 19 mg/g, 104.4 mg/g	Alzheimer’s disease	APP/PS1 mice	↓ Aβ plaque burden, ↑ cortical and hippocampal insulin-degrading enzyme levels, ↑ NGF maturation, ↑ hippocampal neurogenesis
Tzeng et al. (2018)	EA, ES	Alzheimer’s disease	APP/PS1 mice	↑ cortical and hippocampal insulin-degrading enzyme levels, ↑ NGF maturation, ↑ hippocampal neurogenesis. EA; ↓ Aβ production
Wu et al. (2023) ^a^	HEME – [EA]: 5 mg/g	Oxidative stress induced by tBH	tBH treated ELAV-SCA3tr-Q78 transgenic *Drosophila*	↑ lifespan, ↓ apoptosis via downregulation of p53 and NF-kB
Yang et al. (2020) ^a^	EA, ES	Neuropathic pain, neuro-inflammation	C57BL/6 mice with L5 spinal nerve ligation	ES was able to induce better pain-relieving effects when compared to EA

^a^ Indicates studies included in both *in vitro* and *in vivo* results.

Abbreviations: Aβ, amyloid-beta; APP, amyloid precursor protein; BDNF, brain-derived neurotrophic factor; EA, erinacine A; EC, erinacine C; ES, erinacine S; GSK-3β, glycogen synthase kinase-3, beta; GLT-1, glutamate transporter-1; HE, *hericium erinaceus*; HEM, HE, mycelia; HEME, HEM, erinacine extracts; Hip, hippocampus; ICR, institute of cancer research; LPS, lipopolysaccharide; MPTP, 1-methyl-4-phenyl-1, 2, 3, 6-tetrahydropyridine; NF-κB, nuclear factor kappa-light-chain-enhancer of activated B cells; NGF, neuron growth factor; Nrf2, nuclear factor erythroid 2–related factor 2; PS1, presenilin 1; SAMP8, Senescence Accelerated Mouse-Prone 8; SD, sprague dawley; TBI, traumatic brain injury; tBH, *tert*-butyl hydroperoxide; tHI, transient hypoxia-ischemia.

**FIGURE 2:**
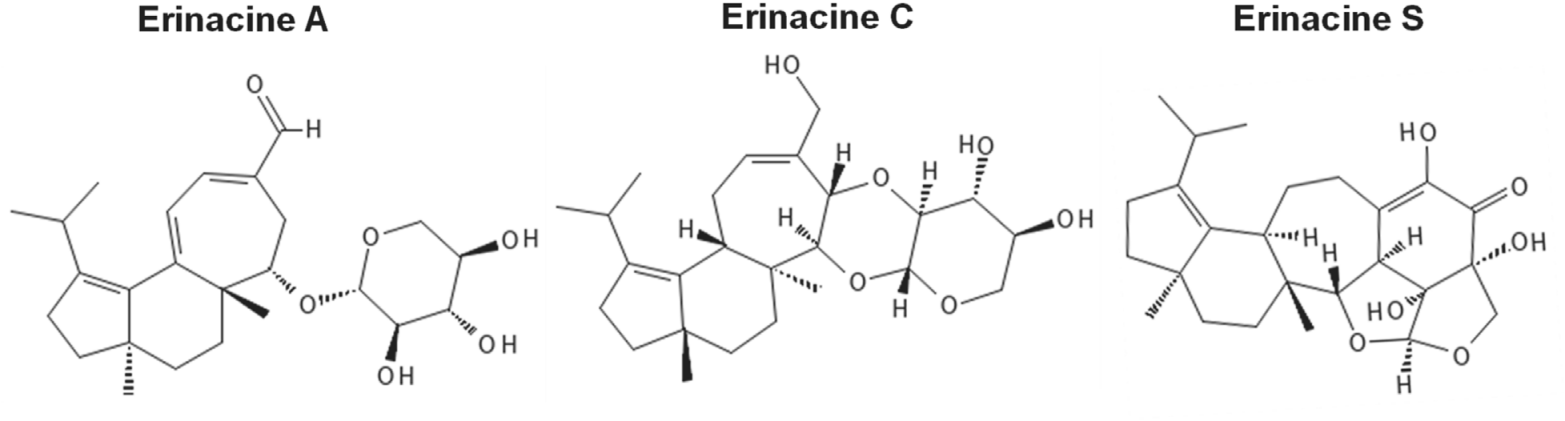
Molecular structures of erinacine A, C and S. Erinacine structures were drawn with the online software PubChem Sketcher, v2.4 (https://pubchem.ncbi.nlm.nih.gov//edit3/index.html), accessed on 24 November 2024 (Ihlenfeldt et al., 2009).

The studies described in this systemic review used various methodologies for the extraction of erinacines. Typically, erinacines extracts derived from *H. erinaceus* mycelia are cultured on nutritive media (e.g., yeast salt medium) using solid state agar or shaking liquid cultures (Gonkhom et al., 2022). Solvent systems used for erinacine extraction from *H. erinaceus* mycelia include acetone (Rupcic et al., 2018), and ethyl acetate (Shen et al., 2015) but ethanol (e.g., 75%–95% ethanol) is most often used (Chen et al., 2016; Liu et al., 2024). This includes the reflux of successive ethanolic extraction of mycelia containing erinacines A, C, and S (Tsai-Teng et al., 2016). The molecular structures of these erinacines are provided in Figure 2.

Typically, the isolation of erinacines from mycelial extracts is achieved by partitioning against solvents such as ethyl acetate, followed by the subsequent resolution and fractionation on chromatographic media such as silica gel and size exclusion resins (Kawagishi et al., 1994; Chen et al., 2016). Other chromatographic strategies used for erinacine purification include preparative high pressure liquid chromatography (HPLC) or flash chromatography (Rupcic et al., 2018). For many of the studies assessed in this review, the erinacine profiles within HEM were often assessed through HPLC or ultra performance liquid chromatography. The identity of erinacines was confirmed by co-chromatography with authentic standards. In addition, erinacines were identified by mass spectrometry and/or nuclear magnetic resonance spectroscopy. It is worth noting that the range of erinacine A concentrations that were detected within HEM varied from 5 to 19 mg/g of freeze-dried or fresh mycelia (Tsai-Teng et al., 2016; Lee et al., 2021; Li et al., 2021; Wu et al., 2023), although Hsu C.-H. et al. (2023), identified a much lower concentration at 30 μg/g dry weight of HEM. Conversely, Tsai-Teng et al. (2016) determined that HEME contained nearly an order of magnitude more erinacine A (i.e., 104.4 mg/g) by comparison. Moreover, this group used HEME that contained 15 times more erinacine A than erinacine C and erinacine S which were each detected at approximately 1 mg/g of mycelial powder.

### *In vitro* studies

#### Models and treatments

Studies only evaluated the acute effects of a single treatment with erinacines or erinacine-containing mycelia (n = 14) and were conducted most often using the PC12 neuron-like cell line (n = 4) or with BV-2 microglial cells (n = 5). Studies also used primary neuronal cell cultures (n = 5) or other neuron-based cell lines such as mouse N2a cells (n = 2) and human neuroblastoma SH-SY5Y cells (n = 2). Molecular and cellular outcomes were assessed most frequently using Western blotting (n = 10) to identify protein expression, or through various polymerase chain reaction (PCR) techniques (n = 8) to quantify gene expression in the cells.

Cells were treated with erinacines at concentrations ranging from 0.01 ng/mL to 25 μg/mL, with erinacine A generally delivered at greater concentrations than erinacine C and erinacine S. *In vitro* HEME was administered from 1.25 to 5 μg/mL and with an erinacine A concentration of 5 mg/g of HEME. There was a general concentration-response trend identified across all groups, with higher concentrations of erinacines and HEME eliciting a stronger cellular response. However, administration of erinacines A, C, or S to SH-SY5Y cells at very high concentrations (>10 μg/mL) decreased cell viability, with as little as 5 μg/mL erinacine S affecting viability in a single study, indicating a biological efficacy window *in vitro* (Yang et al., 2020; Lin et al., 2024).

### Molecular and cellular alterations

#### Anti-inflammatory, antioxidant and neuroprotective effects

When looking at the acute effects of erinacines and/or *H. erinaceus* mycelia in inflammatory and/or disease models, neuroprotective effects were commonly observed. In studies of neuroinflammation models (n = 7), both erinacine A and HEME protected N2a neuronal cells from lipopolysaccharide (LPS)-exposed BV-2 conditioned medium-induced cell death through the suppression of c-Jun N-terminal kinase (JNK) and nuclear factor kappa-light-chain-enhancer of activated B (NF-κB) activation (Lee et al., 2022). Erinacine A and erinacine C inhibited the expression of proinflammatory factors, such as tumor necrosis factor-α (TNF-α), interleukin-6 (IL-6), and inducible nitric oxide synthase (iNOS), implicated in the activation of glial cells (Wang et al., 2019; Lee et al., 2022; Wei et al., 2023). Furthermore, erinacine A ameliorated MPP+ -induced apoptosis and degeneration of dopaminergic neurons in a N2a cell model of Parkinson’s Disease. This neuroprotective effect was proposed to be the result of an induction of anti-apoptotic factors such as activated kinase 1 (PAK1), protein kinase B (AKT), lim domain kinase 2 (LIMK2), mitogen-activated protein kinase, and cofilin. This action was also mediated by the inactivation of several proteins that play key roles in pro-apoptotic pathways, such as TNF-α, or in the promotion of oxidative or endoplasmic reticulum stress (Lee et al., 2020).

In primary cortical glia-neuron co-cultures, erinacine A improved neuronal and astrocyte survival and preserved astrocyte glutamate homeostasis in response to oxygen-glucose deprivation via the accumulation of glutamate transporter 1 protein levels (Hsu et al., 2022). The study attributed the inhibition of the NF-κB and AKT signaling pathways as the targets of erinacine A-mediated neuroprotection. In contrast to the findings of Lee et al. (2022), Hsu et al. (2022) found no effect of erinacine A on alterations in the JNK, or ERK1/2 and p38/mitogen activated protein kinase (MAPK) signaling pathways. A key role for reduced NF-κB signaling in neuroprotection was also observed following HEME treatment in a *tert*-butylhydroperoxide-induced oxidative stress human neuroblastoma cell model (Wu et al., 2023). Erinacine S induced neurogenesis in both cortical and dorsal root ganglion primary mouse neuron cultures through the enhanced expression of genes encoding for the metabolism of the neurosteroids pregnenolone and progesterone (Lin et al., 2023).

In whole-brain mixed glia co-cultures following LPS insult, erinacine C induced the expression of the transcription factor nuclear factor erythroid 2-related factor 2 (Nrf2), which was correlated with increased protein expression of BDNF and other antioxidant enzymes (Lee et al., 2024). Similarly, in LPS-treated BV-2 microglial cells, erinacine C increased the abundance of nuclear antioxidant/anti-inflammatory Nrf2/heme oxygenase-1 (HO-1) protein (Wang et al., 2019).

#### Neurotrophic effects

Under normal conditions, acute treatments with erinacine A, erinacine C, or erinacine S induced neurite-outgrowth of PC12 neurons in the presence of NGF, and also induced NGF release from astrocytic cells in 1321N1 cell models (Rupcic et al., 2018; Wei et al., 2023). The magnitude of neurite outgrowth was increased when cells were co-treated with erinacines and NGF, compared to NGF alone (Wei et al., 2023). Notably, erinacines did not stimulate NGF synthesis or neurite outgrowth in PC12 cells in the absence of the differentiation inducer NGF. This suggests that erinacine A-induced neurogenesis may be the result of elevated NGF potency as opposed to elevated expression of NGF. However, in primary cortical neuron cultures, erinacine A potentiated NGF-induced neurite outgrowth and was protective against neuronal cell death in the absence of NGF, suggesting erinacine A may serve to acutely mimic the action of neurotrophins (Zhang et al., 2017). NGF-induced neurogenesis of differentiated primary cortical neurons upon treatment with erinacine A was mediated by the TrkA receptor and was extracellular signal-related kinase 1/2 (ERK1/2)-dependent (Zhang et al., 2017). Erinacine A, erinacine S, or HEME stimulated the differentiation of precursor cells into mature oligodendrocytes and enhanced myelination during the development of dissociated cells (Huang et al., 2021). However, erinacine A was a more potent stimulator of these effects as compared to erinacine S, suggesting that the oligodendrocyte maturation properties induced by HEME were most likely due to the action of erinacine A (Huang et al., 2021). Interestingly, only erinacine C increased BDNF expression in primary mixed-glia cultures and astrocytic cell models (Rupcic et al., 2018; Rascher et al., 2020; Lee et al., 2024).

#### Summary

*In vitro* studies on *H. erinaceus* mycelia and their derived erinacines focused on their effects in neuronal and glial cell cultures, using techniques like protein and gene expression analysis. Both *H. erinaceus* mycelia and erinacines promoted neurogenesis, protected against inflammation and oxidative stress, and supported cell survival. However, individual erinacine compounds were observed to have differential effects in certain cellular processes compared to others within the same erinacine family.

### *In vivo* studies

#### Treatments

Erinacines investigated *in vivo* included erinacine A in nine studies, erinacine S in four studies and erinacine C in two studies. Erinacines were administered through oral gavage at doses ranging from 2.6 mg/kg to 30 mg/kg per day, whereas a smaller range of 1 mg/kg to 10 mg/kg per day erinacines were injected intraperitoneally. *H. erinaceus* mycelia was administered *in vivo* as HEME (n = 3) and HEM (n = 4). Doses of HEME were largely based on the original dry weight of HEM prior to extract production. No trends were observed in the studies with respect to the dosage regimen between HEM and HEME. Doses of *H. erinaceus* for some studies were based on the recommended human intake level and utilization of a 100-fold lower concentration (–1 mg/day of dry HEM). Among the *in vivo* studies, *H. erinaceus* was administered orally at a range of 75–1,000 mg/kg per day, however most studies used a dose within the 50–300 mg/kg per day range. *H. erinaceus* was administered daily for a minimum of 9 days and up to 13 weeks, with most studies using a 30-day administration timeline. No studies evaluated the impacts of acute erinacine or *H. erinaceus* mycelium treatments.

#### Behavioural studies

Of the studies examined *in vivo* (n = 16), nine evaluated behavioural outcomes; five studies assessed erinacine effects, and four studies investigated HEM/HEME effects. Erinacine A was examined in three studies, whereas erinacine S and erinacine C were examined in one study each. Using the rotarod test to evaluate motor function in mice, systemically administered erinacine A (1 mg/kg) alleviated MPTP-induced motor coordination disruption and balance (Lee et al., 2020). Motor improvements following daily oral administration of erinacine A (5.0 mg/kg) for 6 weeks were also reported for amphetamine-injected LPS-induced Parkinson’s disease models (Lee et al., 2022). Similar effects on alleviating motor deficits in the beam walking test were observed following erinacine C treatment in traumatic brain injury (TBI)-induced rats (Lee et al., 2024). In tail suspension test-induced sleep disrupted mice, chronic administration of HEM (150 mg/kg, containing 7.2 mg/g erinacine A, and 3.35 mg/g erinacine C), promoted exploratory behaviour and lowered anxiety-like behaviour in the open field test (Li et al., 2021). However, one study determined that open field associated-locomotor activity was unchanged in stressed mice after a chronic oral administration of HEME (300 mg/kg containing erinacine A at 5 mg/g of HEME), although antidepressant-like effects were present (Chiu et al., 2018). The potential for depression- and/or anxiety-like relief by oral HEM for 9 days was established in a model of continuous sleep disturbance (Li et al., 2021). Results from rodent studies that evaluated anxiety showed increased exploratory activity and reduced anxiety-like behaviour in response to HEM/HEME (Chiu et al., 2018; Li et al., 2021). Erinacine A and HEM also reduced deficits in burrowing and nesting tasks, which are often used to evaluate activities of daily living skills, a sign of natural instinctive behaviour (Tsai-Teng et al., 2016; Tzeng et al., 2018).

With respect to aspects of cognition, erinacine A improved spatial learning and memory in rodent models of AD as evidenced by a reduced escape latency in the Morris water maze task (Tzeng et al., 2018). Associative learning and memory were also enhanced in the passive avoidance task and active shuttle avoidance test after long-term daily HEM (431 mg/kg containing 5 mg/g erinacine A) administration in an accelerated aging mouse model (Lee et al., 2021). Additionally, moderate benefits of erinacine A pretreatment on scores in neurological deficit assessments were observed in a brain ischemia mouse model (Hsu et al., 2022). Moreover, erinacine A ameliorated grip strength deficits following ischemia, but did not lead to increases in basal levels of grip strength (Hsu et al., 2022). Interestingly, following spinal nerve injury, erinacine S promoted an analgesic suppression of neuropathic pain in the von Frey test (Yang et al., 2020).

### Molecular and cellular alterations

#### Models and analyzed tissue

*In vivo* studies described neuroprotective effects of erinacines and *H. erinaceus* mycelia in a variety of rodent models through the reduction of neuroinflammation and/or oxidative stress. The most frequently analyzed brain regions were the cerebral cortex (n = 7) and hippocampus (n = 5), followed by the striatum (n = 3) and cerebellum (n = 1). Across all studies, the most often used methodologies (n = 15) were protein quantification by Western blotting, enzyme-linked immunosorbent assay, and immunohistochemistry within brain tissues. Only one study included both male and female animals that were grouped and analyzed independently. A total of 11 studies used exclusively males, and three studies selectively used females. Of the three studies that employed females, all used APP/PS1 transgenic mice, as the female sex appears more susceptible to the induced pathology.

#### Anti-inflammatory, antioxidant and neuroprotective effects

Sub-chronic administration was evaluated in four studies, where single erinacine compounds or HEME were administered daily for five to 7 days by intraperitoneal injection or oral gavage (Lee et al., 2014; Lee et al., 2020; Lee et al., 2024; Hsu et al., 2022). A single rat study analyzed the separate effects of pretreatment with erinacine A and HEME on preventing brain injury during ischemia (Lee et al., 2014). This study revealed that erinacine A prevented neuronal cell death. Moreover, erinacine A effects were mediated through the scavenging of endoplasmic reticulum stress-stimulated free radicals, in addition to inhibiting inflammation via inactivation of iNOS and MAPK, and a reduction in the pro-apoptotic C/EBP homologous protein. In addition, neuroprotective effects of systemic erinacine C were apparent when administered to mice for 6 days following TBI (Lee et al., 2024). This effect was mediated by enhanced activation of the Nrf2/superoxide dismutase type 1 (SOD1) pathway. Increased levels of phosphorylated cAMP response element binding (CREB) protein, in addition to thioredoxin reductase, a key component of the thioredoxin (Trx) neuroprotective pathway, were also observed (Lee et al., 2024). Nrf2 levels were also elevated by erinacine A in the retina and optic nerve of rats following traumatic optic neuropathy, and it was proposed that activation of the Nrf2/HO-1/SOD1 antioxidative stress pathway led to reduced inflammation and apoptosis (Hsu C.-L. et al., 2023). Together these findings suggest that the Nrf2 pathway may be important for the nootropic effects of the erinacines. Studies conducted in healthy rodents showed that erinacine A and erinacine S, promoted the production of myelin basic protein and the maturation of cerebellar oligodendrocytes in neonatal rats, whereas erinacine C had no effect (Huang et al., 2021).

Multiple studies used rodent model systems to elucidate the therapeutic potential of erinacines in neurodegeneration (n = 5). Studies utilizing transgenic APPswe/PS1dE9 AD model mice (n = 3) determined that erinacine A, erinacine S, or HEME administration reduced amyloid-β plaque formation in the cerebral cortex while elevating levels of insulin degrading enzyme, a protein believed to play a significant role in the clearance of amyloid-β (Chen et al., 2016; Tsai-Teng et al., 2016; Tzeng et al., 2018). However, Tzeng et al. (2018) established that erinacine A was more effective than erinacine S at inhibiting amyloid-β accumulation by reducing insoluble amyloid-β and the C-terminal fragments of amyloid processing protein. Interestingly, HEM that was enriched in erinacine A was more effective than HEME at reducing the brain soluble forms of amyloid-β_1-42_, whereas HEME was slightly more effective at decreasing the number of amyloid-β_10_-stained plaques, increasing the level of insulin degrading enzyme, and enhancing hippocampal neurogenesis (Tsai-Teng et al., 2016). Erinacine A pretreatment of LPS-induced, or (1-methyl-4-phenyl-1,2,3,6-tetrahydropyridine) MPTP-induced, Parkinson’s Disease model rodents reduced dopaminergic neurotoxicity and protected against microglia-mediated neuroinflammation through the reduction of pro-inflammatory cytokine expression and reactive oxygen species production in the midbrain (Lee et al., 2020; Lee et al., 2022). Moreover, erinacine A treatment ameliorated dopaminergic degeneration in the striatum and substantia nigra of MPTP model mice by reversing the MPTP-induced decrease in pro-survival signaling (Lee et al., 2020). The sole long-term *in vivo* study analyzed the impact of sub-chronic HEM doses in an accelerated aging mouse model, which revealed similar reductions in oxidative stress and lower amyloid-β plaque aggregation as was seen when using sub-chronic doses (Lee et al., 2021). Notably, this was the one study that evaluated sex differences, but no sex-specific effects were observed (Lee et al., 2021).

#### Neuromodulator and neurotrophic effects

Erinacine A increased microglial and neuronal survival while preserving astrocytic glutamate machinery in a cerebral ischemic mouse model, with increased expression of glutamate transporter 1 and similar glutamate homeostatic regulators within cells (Hsu et al., 2022). HEME ameliorated stress-induced cellular changes in mice. For example, stress-induced decreases in hippocampal dopamine, serotonin, and norepinephrine, as well as alterations in plasma IL-6 and TNF-α were ameliorated by the daily administration of HEME (300 mg/kg containing erinacine A at 5 mg/g of dry weight) for 4 weeks (Chiu et al., 2018). Furthermore, HEME supplementation increased hippocampal BDNF levels, activated the AKT signaling pathway to inhibit glycogen synthase kinase-3β (GSK-3β) activity, and induced antidepressant-like behavioral effects (Chiu et al., 2018). Increased BDNF protein expression was evident in whole brain samples following treatment with 150 g/kg HEM (containing 7.2 mg/g erinacine A) to tail suspension test-induced sleep disrupted mice for 9 days (Li et al., 2021). An elevation in hippocampal AKT activity was evident in studies that examined erinacine A and HEME (Chiu et al., 2018; Lee et al., 2020). HEME ameliorated repeated restraint stress or 3-acetylpyridine-induced decreases in monoamine dopamine and serotonin levels (Chiu et al., 2018).

#### Summary

Studies on *H. erinaceus* mycelia and erinacines explored their effects on behavioral and molecular outcomes in rodent models. Behaviorally, these compounds showed similar benefits in motor function, cognition, and emotional regulation, with potential applications in neurodegenerative diseases, anxiety, and pain management. Molecular and cellular findings revealed neuroprotective effects, that involved reductions in inflammation, oxidative stress, and amyloid-β accumulation, alongside enhanced neurogenesis and neuronal survival across both erinacine and *H. erinaceus* mycelial administrations, and with certain erinacines, such as erinacine S, exhibiting novel neurologic effects, particularly as an analgesic.

## Discussion

Although the health benefits associated with *H. erinaceus* are documented to some degree in the scientific literature, there is less information on the contribution of the individual active molecules. As described in this review, *in vitro* and *in vivo* studies to date that have examined the short- and long-term effects of the erinacines, particularly erinacine A and C, as well as *H. erinaceus* mycelia on cellular and behavioral outcomes were evaluated.

Evidence indicates that erinacines or *H. erinaceus* mycelia have neuroprotective properties that are elicited by the upregulation of cell survival factors and the downregulation of oxidative stress and neuroinflammatory pathways. Similar effects were apparent when comparing the molecular and cellular responses to the administration of single erinacine molecules to those of mycelial extracts. For example, the administration of erinacine A, C, and S, as well as those of HEM/HEME, reduced the abundance of pro-inflammatory cytokines such as IL-6, TNF-α, and IL-1β (Chiu et al., 2018; Wang et al., 2019; Yang et al., 2020; Lin et al., 2024). Erinacine A inhibited TNF-α in an astrocyte cell model of neuroinflammation, but not in microglia, although an effect on iNOS signaling selectively in microglia was evident (Lee et al., 2022). Accordingly, *in vivo* treatment of HEM containing 5 mg/g of erinacine A inhibited the induced protein expression of cortical and hippocampal iNOS in response to LPS-induced neuroinflammation (Lee et al., 2021). This effect was also observed across studies of erinacine A *in vivo* (Lee et al., 2014; Lee et al., 2022; Hsu C.-L. et al., 2023) and erinacine C *in vitro* (Wang et al., 2019), whereas this was not investigated with erinacine S. Other similarities in therapeutic actions were observed between erinacine A and S when compared to HEME (containing 104.4 mg/g of erinacine A) and HEM (containing 19.9 mg/g erinacine A) in neurodegenerative disease models. Each treatment was able to decrease the burden of Aβ plaques, increase the levels of insulin degrading enzyme and induce hippocampal neurogenesis while alleviating behavioural deficits observed in the nesting task (Chen et al., 2016; Tsai-Teng et al., 2016; Tzeng et al., 2018). Interestingly, it was observed that HEME, which was higher in erinacine A concentration was more potent at reducing amyloid-β plaque burden as compared to HEM (Tsai-Teng et al., 2016). This indicates that erinacine A may play a crucial role in the mitigation of amyloid-β accumulation observed in mycelial treatments. Studies using neurodegenerative disease models also showed increased activity of NF-kB, a protein involved in the induction of pro-apoptotic factors (Lanzillotta et al., 2015). Thus, the observed reduction of NF-kB activity by HEME and erinacines may be a key mediator underlying the reported neuroprotective effects across the various treatments (Wang et al., 2019; Lee et al., 2022; Wu et al., 2023).

Both erinacines and *H. erinaceus* mycelia reduce the production of reactive oxygen species. The suppression of oxidative stress and inflammation were partially mediated by antioxidant-related processes associated with Nrf2, HO-1, and SOD, which act to sequester free radical production and hence inhibit the activation of pro-inflammatory signaling pathways. Nrf2 is a transcription factor that can bind to promoter regions of genes that are essential for antioxidative, cell survival, and cell proliferation signaling, such as *BDNF, CAT, SOD1* and *TrxR* (Hsu C.-L. et al., 2023; Lee et al., 2024). In rodents, Nrf2 protein levels were elevated in optic nerve and cortical samples after administration of erinacine A or erinacine C (Hsu C.-L. et al., 2023; Lee et al., 2024). Conversely, mouse hippocampal Nrf2 levels were lower *in vivo* following the administration of erinacine-free *H. erinaceus* primordium extracts and, were unaffected when HT22 hippocampal neurons and BV2 microglia were exposed to an erinacine-free *H. erinaceus* fruiting body extract *in vitro* (Kushairi et al., 2019; Roda et al., 2023) suggesting an erinacine-specific effect on Nrf2. Additionally, both erinacine A and HEM protected dopaminergic neurons in models of neurodegeneration and neuroinflammation (Lee et al., 2020; Lee et al., 2022; Li et al., 2021). Conversely, the preservation and elevation of dopaminergic activity was also evident following the *in vivo* administration of fruiting body or mycelia, suggesting erinacines alone are not responsible for the neuromodulatory effects observed in *H. erinaceus* administration (Chiu et al., 2018; Lee et al., 2020; Chau et al., 2023).

*In vivo,* both HEME and HEM that contained a high concentration of erinacine A elevated expression of NGF in specific brain regions such as the hippocampus (Tsai-Teng et al., 2016), although this occurred selectively in astrocytes (Zhang et al., 2017; Rupcic et al., 2018; Rascher et al., 2020). *In vitro* studies using primary cortical neurons suggest that erinacine A can potentially mimic, at least in part, the neurogenic actions of NGF (Zhang et al., 2017). Previous studies have determined that erinacines induce astrocytic NGF protein expression *in vitro* (Kawagishi et al., 1994; Mori et al., 2008). NGF-dependent neurogenesis was mediated by TrkA receptor activation and partially reliant on ERK1/2, a downstream effector of TrkA and TrkB that is involved in promoting axonal growth and neuronal survival (Mitchell et al., 2012; Zhang et al., 2017). HEME or erinacine A-meditated cell survival and growth through both TrkA and TrkB activation may also be mediated by the downstream PI3K/AKT pathway, which regulates essential cellular processes, inhibits apoptosis, and can increase downstream levels of CREB (Li et al., 2015; Chiu et al., 2018; Lee et al., 2020). CREB can be activated through either p38, PI3K/AKT, or ERK signaling, and is critical for increased long-term potentiation (LTP), a process associated with learning and memory, and thus its implication as a key player in *H. erinaceus* and erinacine-mediated amelioration of cognitive and behavioural deficits in disease models (Ortega-Martínez, 2015). Direct evidence of CREB activation was observed *in vivo* after erinacine C administration to TBI-model rats (Lee et al., 2024), however erinacine A and S were not directly investigated. There is evidence that the inhibition of GSK-3β, a downstream target of AKT involved in LTP impairment, provides therapeutic benefits in depression and AD models (Llorens-Marítin et al., 2014). Thus, the reported decrease in GSK-3β observed following administration of HEME enriched in erinacine A (Chiu et al., 2018) may also play a significant role in learning and memory improvements.

Of the analyzed *in vivo* studies, there was widespread similarity in the behaviours observed in response to administration of the erinacines or *H. erinaceus* mycelia, with both inducing improvements in motor functioning, learning and memory, and/or natural instinctive behaviour in the various models examined. However, there was variation in the potency of the cellular effects of the individual erinacines. Although erinacine A was the focus of most of the studies, the *in vivo* effects of erinacine S and erinacine C treatment were also investigated in some reports. The erinacines studied had some similarities in their biological impacts. Amelioration of neuroinflammation by erinacines A, C and S was mediated through the reduced activation of glial cells and decreased release of pro-inflammatory cytokines, and likely involve TrkA receptors (Lee et al., 2020; Lee et al., 2022; Rascher et al., 2020). In addition to NGF/TrkA, HEM administration to healthy mice can also induce TrkB activation through increased abundance of BDNF, an effect shown to involve erinacine C, but not erinacine A (Rupcic et al., 2018). In stressed mice, suppressed BDNF levels in whole brain or hippocampus were not only normalized, but elevated beyond that observed in non-stressed controls following HEM administration (Chiu et al., 2018; Li et al., 2021). Importantly, both *H. erinaceus* ethanolic and aqueous fruiting body extracts increase expression of BDNF protein in models of stress, indicating that erinacines are not the only BDNF-inducing compound found within *H. erinaceus* (Chong et al., 2021; Chau et al., 2023). Erinacine A and erinacine S were also implicated in their ability to stimulate maturation of oligodendrocytes and myelination in healthy models, whereas erinacine C was unable to potently do so (Huang et al., 2021). Interestingly, erinacine A and erinacine S differed in their therapeutic efficacy in the AD mouse model studies. Erinacine A was more potent at inhibiting amyloid-β plaque formation than erinacine S, particularly within the plaque core formation stage and the production of amyloid-β (Tzeng et al., 2018). Conversely, in a spinal nerve ligation mouse model, erinacine S had a greater ability to suppress ATP-induced purinoceptor activation and produce greater pain alleviating effects than erinacine A (Yang et al., 2020). Given the limited overlap between the erinacine studies, there were many effects attributed to single erinacine molecules that were not investigated using others. For example, erinacine A was seen to maintain astrocytic glutamate homeostasis through preservation of GLT-1 function and to increase expression of excitotoxicity protection machinery in a cerebral ischemia model (Hsu et al., 2022) whereas erinacine S was shown to increase accumulation of neurosteriods in primary cortical mouse neurons (Lin et al., 2023). Taken together, these findings suggest that although erinacines share a cyathane backbone within their chemical structure, they can vary in their potency as well as in their ability to alter select cellular functions. A general trend within the studies was that *H. erinaceus* or erinacines exerted their effects in a dose-dependent manner, although some exceptions have been reported. Evidence of a a therapeutic effect of *H. erinaceus* fruiting body extract was apparent in certain disease models, such as in temporal lobe epilepsy model mice (Jang et al., 2019), suggesting there may be an optimal dose range for *H. erinaceus*, as well as for the erinacines. *In vitro* evidence also suggests treatment with erinacines at high concentrations can negatively affect cell viability (Lin et al., 2024). Interestingly, erinacine S decreased cell viability more potently than erinacine A or C when administered at the same concentration to BV-2 microglia and SH-SY5Y neuron co-cultures, suggesting the erinacines may have different toxic dose ranges (Lin et al., 2024). However, this was unsupported by *in vivo* studies, where no dose of erinacines – oral or systemic – decreased the abundance of cell survival markers. The cell viability effects reported *in vitro* may be a possible reflection of the lack of cellular complexity in these models.

### Future considerations

It is noteworthy that sex effects were, for the most part, not evaluated in the *in vivo* studies. A single study considered the sex-dependent effects of erinacines or *H. erinaceus* (Lee et al., 2021), whereas the vast majority of studies focused on one sex, most often male subjects. This lack of representation of both sexes may lead to missed therapeutic opportunities and lack of identification of potential sex-specific effects, particularly among females that are most often understudied. Indeed, many studies report sex differences in learning and memory, as well as in several disorders and diseases such as autism spectrum disorders, depression, or neurodegenerative disorders (Altemus et al., 2014; Williams et al., 2021; Aggarwal and Mielke, 2023; Fleischer and Frick, 2023).

In addition to the lack of sex inclusion, there is a lack of quantification of the chemical constituents within *H. erinaceus* studies in the general literature. This lack of erinacine quantification led to the exclusion of a large majority of *H. erinaceus* focused studies prior to analysis. This is particularly problematic as numerous factors contribute to the chemical fingerprint of mushrooms, such as the strain used, availability of carbon and nitrogen sources (Dudekula et al., 2020), growth conditions (Corana et al., 2019), as well as the chemical extraction procedure. Strain identification and reporting were not featured across studies. This, together with the lack of consistent reporting of growth conditions, makes it challenging to directly compare the *H. erinaceus* studies as the ratio of compounds would invariably differ between reports. With respect to extraction methods, the lack of standardization across extract formulations presents a significant limitation. Hot-water, ethanol and methanol-based extraction of mycelium were common approaches, as well as administration of lyophilized crude extracts, each of which would result in differing concentrations of erinacines administered across studies, limiting repeatability and reliability. Additionally, when reporting doses, some studies referred to the dry weight of HEM prior to concentration whereas others referred to the final extract creating confusion for future applications. Taken together, the lack of erinacine concentration reporting given the variability of strain, cultivation, extraction and dosing greatly reduces the translatability and reproducibility of studies using *H. erinaceus* mycelia. Therefore, future studies should establish standardized protocols for batch-to-batch consistency in HEME formulation.

The use of pure compounds may present a logical alternative for therapeutics given the challenge in creating extracts with the same chemical compositions between laboratories or facilities. A more thorough characterization of the neuroprotective and neurogenic pathways induced by erinacines is required as are investigations into the pharmacokinetics and safety profiles of these compounds. Moreover, although the broad neuroprotective effects of erinacine A, C and S have been identified, the actual binding target(s) of the compounds remain unknown. Future studies should attempt to identify the direct receptor and/or targets in which erinacines associate to produce their neuroprotective effects to gain a deeper understanding of their mechanism of action prior to clinical implementation. Future research should assess erinacine A, C or S administration in parallel with HEM or HEME administrations using equivalents of the individual compounds to dose. In this way it would be possible to discern directly how the biological effects of the whole mushroom or mycelium differs from the individual molecule; that is, whether there is an “entourage effect” from the other sparsely studied compounds such as hericerins and erinacerins.

This systematic review highlights the neuroprotective actions of erinacine compounds in *H. erinaceus.* We found that erinacines uniquely activate Nrf2 pathways to promote neuroprotection through various signaling mechanisms, with erinacine C demonstrating promise as a neuroprotective agent by elevating BDNF signaling. Additionally, erinacine S yielded analgesic effects for neuropathic pain, underscoring the compound’s potential relevance to public health challenges like neurodegenerative diseases and chronic pain management. Through their observed neuroprotective and neurotrophic effects, associated with reductions in neuroinflammation and oxidative stress, evidence supports the use of erinacines and erinacine-containing compounds as potential therapeutics in neurodegenerative diseases and in preventing overall neurocognitive decline. Furthermore, given the apparent high tolerability observed with HEM/HEME administration, alongside the low cost and accessibility, the study of *H. erinaceus* for broader therapeutic applications, such as for TBI, anxiety disorders, or major depressive disorder, is recommended.

## Glossary

AD: Alzheimer’s Disease
AKT: Protein kinase B
APP: Amyloid precursor protein
ATP: Adenosine triphosphate
BDNF: Brain-derived neurotrophic factor
CREB: cAMP response element-binding protein
CTX: Cortex
C/EBP: CCAAT-enhancer-binding proteins
EA: Erinacine A
EB: Erinacine B
EC: Erinacine C
EF: Erinacine F
EL: Erinacine L
ERK1/2: Extracellular signal-regulated kinase 1/2
ES: Erinacine S
EZ1: Erinacine Z1
EZ2: Erinacine Z2
GLT-1: Glutamate transporter 1
GSK-3β: Glycogen synthase kinase-3 beta
HE: *Hericium erinaceus*
HEM: *Hericium erinaceus* mycelia
HEME: *Hericium erinaceus* mycelia extracts
Hip: Hippocampus
**H**_**2**_ **O**_**2**_: Hydrogen peroxide
HO-1: Heme oxygenase-1
ICR: Institute of Cancer Research
IFN-γ: Interferon-gamma
IL-6: Interleukin-6
iNOS: Inducible nitric oxide synthase
JNK: c-Jun N-terminal kinase
LIMK2: LIM domain kinase 2
LPS: Lipopolysaccharide
MAPK: Mitogen-activated protein kinase
MPP+: 1-Methyl-4-phenylpyridinium
MPTP: 1-Methyl-4-phenyl-1,2,3,6-tetrahydropyridine
NF-κB: Nuclear factor kappa-light-chain-enhancer of activated B cells
NGF: Nerve growth factor
Nrf2: Nuclear factor erythroid 2-related factor 2
NT-3: Neurotrophin-3
OPC: Oligodendrocytes progenitor cell
PAK1: p21-activated kinase 1
PCR: Polymerase chain reaction
PI3K: Phosphoinositide 3-kinase
pCREB: Phosphorylated CREB
PRISMA: Preferred Reporting Items for Systematic reviews and Meta-Analyses
PS1: Presenilin 1
SAMP8: Senescence Accelerated Mouse-Prone 8
SCA3: Spinocerebellar ataxia type 3
SD: Sprague Dawley
SOD1: Superoxide dismutase 1
TBI: Traumatic brain injury
tBT: Tert-butyl hydroperoxide
tHI: Transient hypoxia-ischemia
**TNF**-α: Tumor necrosis factor-alpha
Trk: Tyrosine receptor kinase
TrkA: Tyrosine receptor kinase A
TrkB: Tyrosine receptor kinase B
Trx: Thioredoxin
TrxR: Thioredoxin reductase
